# Role in virulence of phospholipases, listeriolysin O and listeriolysin S from epidemic *Listeria monocytogenes* using the chicken embryo infection model

**DOI:** 10.1186/s13567-017-0496-4

**Published:** 2018-02-06

**Authors:** Juan J. Quereda, Christopher Andersson, Pascale Cossart, Jörgen Johansson, Javier Pizarro-Cerdá

**Affiliations:** 1 Unité des Interactions Bactéries-Cellules, Département de Biologie Cellulaire et Infection, Institut Pasteur, 75015 Paris, France; 2 Institut National de la Santé et de la Recherche Médicale, U604, 75015 Paris, France; 3 Institut National de la Recherche Agronomique, USC2020, 75015 Paris, France; 4 Department of Molecular Biology, Umeå University, 90187 Umeå, Sweden; 5 Laboratory for Molecular Infection Medicine Sweden (MIMS), Umeå University, 90187 Umeå, Sweden; 6 Umeå Center for Microbial Research (UCMR), Umeå University, 90187 Umeå, Sweden; 7 Present Address: Grupo Fisiopatología de la Reproducción, Departamento Producción y Sanidad Animal, Salud Pública Veterinaria y Ciencia y Tecnología de los Alimentos, Facultad de Veterinaria, Universidad Cardenal Herrera-CEU, CEU Universities, Valencia, Spain; 8 Unité de Recherche Yersinia, Département de Microbiologie, Institut Pasteur, 75015 Paris, France; 9 Centre National de Référence “Peste et autres Yersinioses, Institut Pasteur, 75015 Paris, France; 10 Centre Collaborateur OMS de Référence et de Recherche “Yersinia”, Institut Pasteur, 75015 Paris, France

## Abstract

Most human listeriosis outbreaks are caused by *Listeria monocytogenes* evolutionary lineage I strains which possess four exotoxins: a phosphatidylinositol-specific phospholipase C (PlcA), a broad-range phospholipase C (PlcB), listeriolysin O (LLO) and listeriolysin S (LLS). The simultaneous contribution of these molecules to virulence has never been explored. Here, the importance of these four exotoxins of an epidemic lineage I *L.* *monocytogenes* strain (F2365) in virulence was assessed in chicken embryos infected in the allantoic cavity. We show that LLS does not play a role in virulence while LLO is required to infect and kill chicken embryos both in wild type transcriptional regulator of virulence PrfA (PrfA^WT^) and constitutively active PrfA (PrfA*) backgrounds. We demonstrate that PlcA, a toxin previously considered as a minor virulence factor, played a major role in virulence in a PrfA* background. Interestingly, GFP transcriptional fusions show that the *plcA* promoter is less active than the *hly* promoter in vitro, explaining why the contribution of PlcA to virulence could be observed more importantly in a PrfA* background. Together, our results suggest that PlcA might play a more important role in the infectious lifecycle of *L.* *monocytogenes* than previously thought, explaining why all the strains of *L.* *monocytogenes* have conserved an intact copy of *plcA* in their genomes.

**Electronic supplementary material:**

The online version of this article (10.1186/s13567-017-0496-4) contains supplementary material, which is available to authorized users.

## Introduction

Listeriosis, a zoonotic foodborne disease of mammals and birds, is caused by the Gram-positive facultative intracellular bacterium *Listeria monocytogenes.* In humans, listeriosis is characterized by febrile gastroenteritis, meningoencephalitis, abortion and septicemia with a mortality rate of 30% [1]. *L.* *monocytogenes* infections in birds result in focal necrosis of intestine, spleen, liver, kidneys, heart, lungs and air sacks, while meningoencephalitis is uncommon [2, 3].

Four *L.* *monocytogenes* exotoxins have been described to date: PlcA, PlcB, the cholesterol-dependent cytotoxin LLO and the thiazole/oxazole-modified toxin LLS. *plcA, plcB* and *hly* (the gene encoding LLO) are encoded in the *Listeria* Pathogenicity Island 1 (LIPI-1) under the transcriptional control of the PrfA regulator and contribute to escape from the endocytic and secondary vacuoles [1, 4, 5]; traditionally, a predominant role on vacuolar escape has been attributed to LLO and PlcB over PlcA [6]. LLS is a streptolysin S (SLS)-like virulence factor encoded by *llsA* in the *Listeria* Pathogenicity Island 3 (LIPI-3). LLS causes only weak red blood cell hemolysis in vitro and neither is cytotoxic for eukaryotic cells nor confers resistance to phagocytic killing [7]. LLS also behaves as a bacteriocin, being preferentially expressed in the intestine of infected mice and favoring colonization of the intestine by *L.* *monocytogenes* [7, 8].

*Listeria monocytogenes* pathogenesis studies have been mainly performed with the evolutionary lineage II strains EGD-e, EGD and 10403S that possess the LIPI-1 but lack LIPI-3. Interestingly, these lineage II strains have been rarely associated to human disease [9, 10]. On the contrary, a subset of *L.* *monocytogenes* lineage I strains that are frequently associated with human listeriosis outbreaks possess LIPI-3 [7] besides expressing LIPI-1. These lineage I strains have been poorly characterized and no studies to date have addressed the simultaneous impact of LIPI-1 and LIPI-3-encoded toxins on virulence.

The chicken embryo has been recently reported as a reliable, inexpensive and easy to set up infection model for studying *L.* *monocytogenes* pathogenesis and several other bacterial diseases [11–14]. The present study was undertaken to gain deeper insight into the role of the PlcA, PlcB, LLO and LLS exotoxins of the epidemic *L.* *monocytogenes* F2365 strain (responsible for the 1985 California listeriosis outbreak [15]) in chicken embryos infected in the allantoic cavity.

## Materials and methods

### Bacterial strains and cell lines

The bacterial strains used are listed in Table 1. *L.* *monocytogenes* strains were grown at 37 °C in brain heart infusion (BHI) broth in shaking (180 rpm) aerobic conditions. *Escherichia coli* strains were grown in Luria–Bertani (LB) broth at 37 °C in shaking (180 rpm) aerobic conditions. When required, media were supplemented with chloramphenicol 7 µg/mL, erythromycin 1.5 µg/mL or ampicillin 100 µg/mL. The tissue culture cells used in this study were Jeg-3 cells (human epithelial placenta cells; ATCC HTB-36) and HD11 cells (avian macrophage cell line [16]). Cells were maintained in Dulbecco’s modified Eagle’s medium (DMEM) (Gibco) 2 mM Glutamax supplemented with 10% (vol/vol) fetal calf serum (Biowest). Cells were grown at 37 °C with 10% CO_2_.

**Table 1:** **Bacterial strains used in this study**

BUG	Mutation/relevant genotype	Strain	References
1600	wild type	*L.* *monocytogenes* EGD-e	[10]
3012	wild type	*L.* *monocytogenes* 4b F2365	[15]
3673	Δ*plcA*	*L.* *monocytogenes* 4b F2365	This study
3671	Δ*hly*	*L.* *monocytogenes* 4b F2365	This study
4077	Δ*plcB*	*L.* *monocytogenes* 4b F2365	This study
3781	Δ*llsA*	*L.* *monocytogenes* 4b F2365	[8]
3817	pHELP:LLS	*L.* *monocytogenes* 4b F2365	[8]
3651	prfA*	*L.* *monocytogenes* 4b F2365	This study
3702	prfA* Δ*plcA*	*L.* *monocytogenes* 4b F2365	This study
3657	prfA* Δ*hly*	*L.* *monocytogenes* 4b F2365	This study
3703	prfA* Δ*plcB*	*L.* *monocytogenes* 4b F2365	This study
3615	pMAD:prfA* from *L.* *monocytogenes* 4b F2365	*E. coli*	This study
3669	pMAD:*plcA* from *L.* *monocytogenes* 4b F2365	*E. coli*	This study
3667	pMAD:*hly* from *L.* *monocytogenes* 4b F2365	*E. coli*	This study
3670	pMAD:*plcB* from *L.* *monocytogenes* 4b F2365	*E. coli*	This study
4060	pAD-P*hly*-GFP	*L.* *monocytogenes* 4b F2365 inlB corrected	This study
4062	pAD-P*plcA*-GFP	*L.* *monocytogenes* 4b F2365 inlB corrected	This study
4052	pAD-P*hly*-GFP	*E. coli*	This study
4056	pAD-P*plcA*-GFP	*E. coli*	This study

### Mutant construction

To construct the different deletion mutant strains, fragments containing 500 bp DNA flanking the ORFs of *plcA*, *plcB*, and *hly* were amplified by PCR using chromosomal DNA of *L.* *monocytogenes* strain F2365 and cloned into the suicide integrative vector pMAD as previously described [17]. Oligonucleotides used in PCR are listed in Additional file 1. To construct the F2365 PrfA* mutant strain which contains a point mutation (G145S) in PrfA rendering it constitutively active, the *prfA* gene and its flanking regions were amplified by SOEing PCR using genomic DNA from F2365 and the oligonucleotides prfA*-A/prfA*-B and prfA*-C/prfA*-D (Additional file 1). These oligonucleotides introduce a silent mutation in Cys144 (codon TGC to TGT) and a missense mutation in Gly145 changing it to Ser145 (codon GGT to TCT). The PCR fragment generated was inserted into pMAD. Allelic exchange using pMAD was induced as previously described [17], and deletion was confirmed by PCR. All plasmids and strains were confirmed by DNA sequencing.

*Listeria monocytogenes* F2365 carries a nonsense mutation in *inlB* (codon number 34 is TAA) [18]. To facilitate in vitro cell infection and imaging, a mutant strain which contains a functional InlB [point mutation in the codon 34 (TAA to CAA)] was used [7].

### Chicken embryo infections

Chicken embryos were used to assess the virulence of *L.* *monocytogenes* as previously reported [11]. Eggs were incubated for 9 days in an egg incubator set at a temperature of 37.5 °C and a moisture level between 60 and 70% before infection. Overnight cultures of *L.* *monocytogenes* grown in BHI were diluted 100 times into fresh BHI and grown at 37 °C in shaking conditions (180 rpm) until OD_600_ = 0.7. Bacteria were washed in 0.9% (wt/vol) NaCl and diluted in the same NaCl solution to 5 × 10^3^ bacteria/mL. The eggshell was perforated aseptically and 100 μL of the bacterial suspension were inoculated in the allantoic cavity [11]. The openings on the eggs were sealed with paraffin and tape. Infected eggs were returned to the incubator and monitored for viability during 48 h. The presence of blood vessels and embryo movement was used to score the viability of the eggs. All experiments with chicken embryos were performed in compliance with the Swedish animal protection law, under which no specific approval is needed for work performed in avian embryos before day 13.

### Gene expression analysis

Total RNA from overnight cultures (OD_600_ ≈ 3) of *L.* *monocytogenes* grown in BHI at 37 °C in shaking conditions (180 rpm) was prepared as previously reported [19]. RNA integrity was assessed by the RNA Integrity Number (RIN) obtained from the Agilent Bioanalyzer. Only RNA samples with a RIN higher than 9.8 were used. For cDNA library construction, we used 1 µg of total DNA-free RNA and the iScript™ cDNA Synthesis Kit (Bio-Rad) in a thermal cycler using the following protocol: priming during 5 min at 25 °C, reverse transcription during 30 min at 42 °C and finally RT inactivation during 5 min at 85 °C. qPCR and gene expression analysis were performed as previously described [19]. Briefly, qPCR reactions were set in a 10 μL final volume containing the KAPA SYBR FAST qPCR Master Mix (KapaBiosystems), 400 nM of gene specific primers and 5 ng of the cDNA library as template. The reactions were performed in a Bio-Rad iCycler (Bio-Rad) using the following reaction conditions: 1 min at 95 °C; 44 cycles of 2 s at 95 °C and 20 s at 55 °C; dissociation curve of 15 s at 95 °C, 1 min at 60 °C and a progressive temperature increase until 95 °C. Expression levels of the genes of interest were normalized using *gyrA* as an internal standard. Oligonucleotides used in qPCR are listed in Additional file 2. The *n*-fold change of the transcript level was calculated using the ΔΔCt method as already described [19]. Statistical significance was analyzed by using Student’s *t* test. A *P* value of < 0.05 was considered significant.

### Western blot

To analyze LLO expression in the *L.* *monocytogenes* F2365 PrfA* and in the *L.* *monocytogenes* PrfA* Δ*plcA* strains, protein extracts of bacteria were prepared from stationary phase cultures grown overnight in BHI at 37 °C in shaking (180 rpm) conditions. Bacterial pellets were sonicated and denatured samples were run on SDS-PAGE gels (Biorad) and transferred onto PVDF membrane (GE Healthcare) to perform immunoblot. The primary antibodies used were affinity-purified rabbit polyclonal antibody anti-LLO (R176) [20] and rabbit polyclonal antibody anti-EF-Tu (R114) [21]. EF-Tu was used as a control of loaded bacteria. The secondary antibodies were HRP-conjugated anti-rabbit (AbCys). The PVDF membranes were developed by enhanced chemiluminescence using ECL2 (Amersham).

### Hemolysis assay

Hemolysis was assessed by streaking 10 µL of frozen bacterial cultures to isolate single colonies onto Trypcase Soy Agar + 5% Horse Blood (Biomerieux) and incubating for 24 h at 37 °C.

### plcA and hly transcriptional fusions

To fuse the *plcA* and *hly* promoters to a fluorescence-encoding gene, we designed a chimeric construction composed of the *plcA* or *hly* promoters (500 bp upstream of the ATG of the respective gene of *L.* *monocytogenes* F2365) fused with the gene encoding GFP-mut2 [22] (generating P*plcA*-GFP and P*hly*-GFP) and cloned into *Sal*I–*Sma*I-digested pAD vector (generating pAD-P*plcA*-GFP and pAD-P*hly*-GFP). Gene synthesis to construct P*plcA*-GFP and P*hly*-GFP was produced by Genecust (Luxembourg). pAD-P*plcA*-GFP and pAD-P*hly*-GFP were electroporated into *L.* *monocytogenes* F2365 InlB corrected (BUG3824) [7].

### Cell infection and epifluorescence analysis of plcA and hly promoter activity

HD11 and Jeg-3 cell suspensions were seeded in 96-well tissue culture plates and grown for 24 h in an antibiotic-free medium. The *L.* *monocytogenes* strains were grown overnight in BHI, washed in PBS, and diluted in DMEM infection media (1% FBS). Bacterial suspensions were added to the eukaryotic cells at a multiplicity of infection (MOI) of approximately two bacteria per cell and incubated for 1 h. The cells were then washed, and extracellular bacteria were neutralized by adding complete medium containing 40 µg/mL of gentamicin. After incubation for 2 or 6 h, the wells were washed with pre-warmed PBS and cells were lysed in distilled water containing 0.1% TritonX-100. The number of viable intracellular *L.* *monocytogenes* was determined by serial dilution and colony counting on BHI agar plates. These experiments used six technical replicates per bacterial strain and were repeated three times using independent derived clones of each of the strains. Statistical analyses were performed using the Student’s *t* test. For epifluorescence analysis of promoter activity, infected cells were fixed with a paraformaldehyde solution (4% in PBS) for 15 min at room temperature and permeabilized (0.1% Triton X-100 for 3 min in PBS). Cells were then rinsed four times in PBS, incubated with phalloidin conjugated to Alexa 546 and Hoechst for 30 min at room temperature, and rinsed four times in PBS. Samples were examined with a Zeiss Axiovert 135 epifluorescence microscope (Carl Zeiss) associated to a charge-coupled device (CCD) camera. Images were obtained with a × 63 oil immersion objective, and processed with MetaMorph software (Universal Imaging).

## Results

### Contribution of PlcA, PlcB, LLO and LLS to *L.* *monocytogenes* virulence in chicken embryos

**Figure 1:**
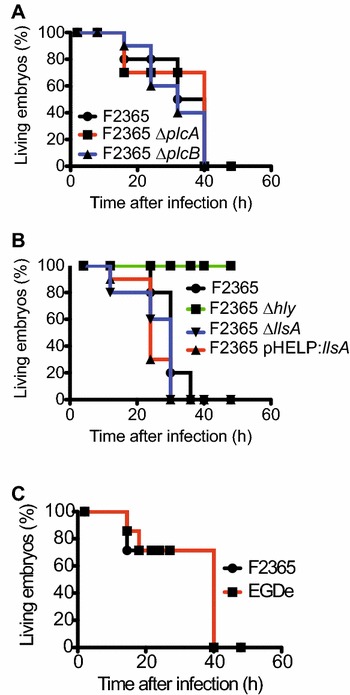
**Survival curves of chicken embryos infected with different strains of** ***L.*** ***monocytogenes***. **A** and **B** Survival curves of 9-day old chicken embryos infected with 5 × 10^2^ *L.* *monocytogenes* from an epidemic lineage I strain (F2365) and the indicated isogenic deletion mutants. The survival of the chicken embryos was followed for 48 h after infection by candling and embryo movement. Data from one representative experiment is shown. At least 2 independent experiments were performed (see Table 2 for details). **C** Survival curves of chicken embryos infected with *L.* *monocytogenes* F2365 and EGD-e strains were performed as described in section **A** of this figure.

The chicken embryo has been shown to be a reliable model to assess the contribution of LLO to virulence upon *L.* *monocytogenes* infection, as deletion of the gene *hly* renders the bacteria fully avirulent [11, 23]. In order to investigate in this avian infection model the role of the four toxins secreted by epidemic *L.* *monocytogenes* strains (PlcA, PlcB, LLO and LLS), we infected eggs with 5 × 10^2^ *L.* *monocytogenes* lineage I F2365 WT strain and similar numbers of the isogenic deletion mutants Δ*plcA*, Δ*plcB,* Δ*hly* and Δ*llsA*. The mean hours until 50 and 100% death of the infected eggs were monitored. The F2365 Δ*plcA* and Δ*plcB* deletion mutants were not attenuated when compared to the WT strain (Figure 1A, Table 2) while the F2365 Δ*hly* deletion mutant was avirulent as previously reported for the *L.* *monocytogenes* lineage II strain EGD-e (Figure 1B, Table 2) [23]. To evaluate the role of LLS, a deletion mutant of the structural LLS gene *llsA* (F2365 Δ*llsA*) [7, 8] and an isogenic mutant that expresses constitutively LLS (F2365 pHELP:LLS) [7, 8] were tested in a chicken embryo survival experiment together with the WT strain. LLS deletion or overexpression did not affect *L.* *monocytogenes* infection of chicken embryos (Figure 1B, Table 2).

**Table 2:** **Mean time until death of chicken embryos**

*L.* *monocytogenes* genotype	BUG strain	Time (h) until 100% death^a^	Time (h) until death of at least 50%^b^	Number of eggs	Number of experiments
EGDe	BUG 1600	41.3 ± 1.35	29.8 ± 10.15	14	2
F2365	BUG 3012	36.5 ± 4.46	31.4 ± 4.35	26	5
F2365 Δ*plcA*	BUG 3673	41.3 ± 1.35	41.3 ± 1.35	13	2
F2365 Δ*hly*	BUG 3671	–	–	21	4
F2365 Δ*plcB*	BUG4077	44.0 ± 0	32.0 ± 0	20	2
F2365 Δ*llsA*	BUG 3781	31.3 ± 1.89	29.3 ± 4.11	15	3
F2365 pHELP::*llsA*	BUG 3817	30.0 ± 0	24.0 ± 0	10	2
F2365 prfA*	BUG 3651	25.4 ± 3.7	17.1 ± 2.6	9	2
F2365 prfA* Δ*plcA*	BUG 3702	–	–	10	2
F2365 prfA* Δ*hly*	BUG 3657	–	–	19	3
F2365 prfA* Δ*plcB*	BUG 3703	41.3 ± 7.9	19.3 ± 4.8	13	2

The number of eggs and number of independent experiments are shown.

^a^Mean time (hours) until complete (100%) death of chicken embryos infected with different *L.* *monocytogenes* strains.

^b^Mean time (hours) until death of at least 50% of the chicken embryos infected with different *L.* *monocytogenes* strains.

The epidemic lineage I strain F2365 is more virulent in an oral mouse infection model than the lineage II strain EGD-e, and this higher virulence is at least partially due to the specific expression of LLS in the intestine of infected mice [8]. Since LLS has no apparent role in virulence in chicken embryos (Figure 1B) and its expression was previously detected only in the intestine of infected animals (and absent in other organs) [7, 8], we speculated that the virulence of the F2365 and EGD-e strains would be similar in chicken embryos infected in the allantoic cavity. To evaluate this hypothesis, we infected eggs with 5 × 10^2^ *L.* *monocytogenes* F2365 or EGD-e strains and monitored for viability during 48 h. 50% of the chicken embryos infected with both strains died ≈ 30 h after infection (Figure 1C, Table 2), confirming that LLS does not play a role in the chicken infection model that bypasses the intestinal stage. This result is relevant as it confirms the absence of a cytotoxic role for LLS [7]. Overall, our results therefore suggest that only LLO plays a major role in virulence in the chicken embryo model.

### Critical role for PlcA in *L.* *monocytogenes* virulence in a PrfA* background

**Figure 2:**
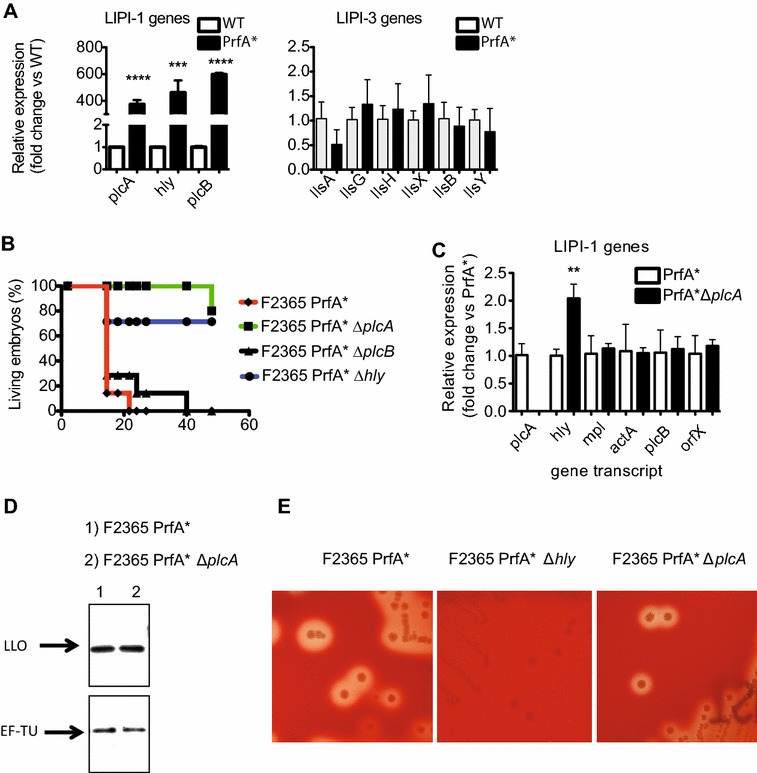
LIPI-1 and LIPI-3 gene expression in a PrfA* background and survival curves of chicken embryos infected with different strains of ***L.*** ***monocytogenes*** F2365 PrfA*. A LIPI-1 and LIPI-3 genes transcript levels monitored by real-time qPCR in *L.* *monocytogenes* F2365 (WT) and F2365 PrfA* strain (PrfA*) strains grown to stationary phase (OD_600_ ≈ 3) in BHI in aerobic shaking conditions. *** = *P* < 0.001; **** = *P* < 0.0001. **B** Survival curves of 9-day old chicken embryos infected in the allantoic cavity with 5 × 10^2^ *L.* *monocytogenes* of the indicated strains. The survival of the chicken embryos was followed for 48 h after infection by candling and embryo movement. Data from one representative experiment is shown. At least 2 independent experiments were performed (see Table 2 for details). **C** LIPI-1 genes mRNA levels examined by real-time qPCR in *L.* *monocytogenes* F2365 PrfA* (PrfA*) and in the *L.* *monocytogenes* F2365 PrfA* Δ*plcA* (PrfA* Δ*plcA*) strains grown to stationary phase OD_600_ ≈ 3 in BHI in aerobic shaking conditions. ** = *P* < 0.01. **D** LLO protein levels detected by Western blot. Levels of a loading control protein, EF-TU, are shown for comparison. Protein extracts of bacteria were prepared from *L.* *monocytogenes* F2365 PrfA* and PrfA* Δ*plcA* strains grown to stationary phase OD_600_ ≈ 3 in BHI in aerobic shaking conditions. **E** Assessment of the hemolytic activity of *L.* *monocytogenes* F2365 PrfA*, F2365 PrfA* Δ*hly* and F2365 PrfA* Δ*plcA* in Trypcase Soy Agar + 5% Horse Blood after incubation at 37 °C during 24 h.

The study of a *prfA* deletion mutant in the EGD-e background indicated previously that the transcriptional activator PrfA is required for full *L.* *monocytogenes* virulence in the chicken embryo model [23]. We evaluated the phenotype associated to constitutive activation of PrfA by using a F2365 PrfA* strain displaying a Gly145Ser substitution in this transcriptional activator that causes constitutive expression of LIPI-1 virulence factors [24]. Since no study has been performed yet to evaluate whether LIPI-3 is regulated by PrfA, we evaluated first the transcript levels of the LLS operon in the F2365 WT and F2365 PrfA* strains. mRNA levels of the LIPI-3 genes were expressed at the same level in the F2365 PrfA* strain compared to the F2365 WT strain (Figure 2A), clearly indicating that PrfA does not control LIPI-3 gene expression. In contrast and as expected, *plcA*, *hly* and *plcB* transcript levels were 400–600-fold higher in the F2365 PrfA* strain compared to the F2365 WT strain (Figure 2A).

To evaluate the role in virulence of PlcA, PlcB and LLO in a PrfA* background, we infected 9 days old eggs with 5 × 10^2^ F2365 PrfA* strain and similar numbers of the isogenic deletion mutants of these virulence factors. The mean hours until 50 and 100% death of the infected eggs were monitored. As expected, the F2365 PrfA* strain killed 50% of the chicken embryos more rapidly than the F2365 WT strain (≈ 17 h vs 31 h, respectively) (Figures 1C and 2B, Table 2). In the F2365 PrfA* strain, LLO was necessary to infect and kill chicken embryos as observed in the F2365 PrfA^WT^ background (Figure 2B, Table 2) [23]. Surprisingly, PlcA was absolutely required for successful killing of chicken embryos in a PrfA* background, while a strain lacking PlcB showed a similar ability as its WT parental strain to kill 50% of the embryos (Figure 2B, Table 2). These results highlight an unsuspected role for PlcA in virulence in a PrfA* background.

*hly* is on the opposite strand of *plcA* in the *L.* *monocytogenes* chromosome. Since 5′ regions often are important for gene expression regulation [25] we studied whether the effect of the deletion of *plcA* in virulence was due to polar effects of the F2365 PrfA*Δ*plcA* mutant. *plcA* deletion in the F2365 PrfA* strain led to a slight increase in the amount of *hly* transcripts whereas it did not alter the transcript levels of the other virulence factors in LIPI-1 (Figure 2C). The increase of *hly* transcript levels in the PrfA*Δ*plcA* mutant could suggest a mechanism of transcriptional interference or promoter competition between *plcA* and *hly*. Nevertheless, we confirmed that the protein levels and the hemolytic activity of LLO in the F2365 PrfA*Δ*plcA* mutant were similar to the WT strain F2365 PrfA* (Figure 2D and E), suggesting that the increased *hly* transcript does not necessarily give increased LLO levels. Our results therefore indicate that LIPI-3 is not regulated by PrfA and that PlcA plays an unsuspected role in virulence when expressed constitutively in a PrfA* background.

### PlcA is poorly expressed but necessary for cell infection in vitro

**Figure 3:**
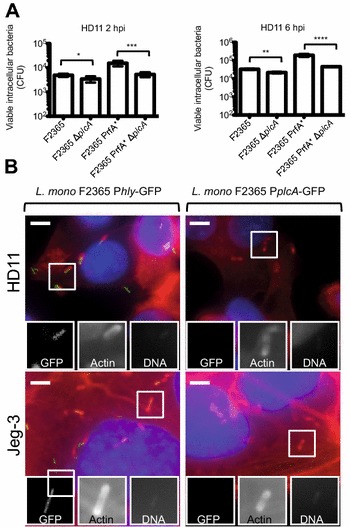
**PlcA role and promoter activity in infection of eukaryotic cells. A** Number of viable intracellular *L.* *monocytogenes* F2365, F2365 ∆*plcA*, F2365 PrfA* and F2365 PrfA* ∆*plcA* in HD11 macrophages. CFU numbers were monitored at 2 h and 6 hpi. Indicated are the number of viable intracellular bacteria determined. Three independent experiments with 6 replicates at each experiment were performed. One representative experiment is shown. Means and standard deviation are shown (*, *P* < 0.05; **, *P* < 0.01; ***, *P* < 0.001; ****, *P* < 0.0001). **B** Fluorescence microscopy to evaluate the promoter activity of *plcA* and *hly* in the *L.* *monocytogenes* epidemic strain F2365. HD11, and Jeg-3 cells were cultured in 96 well plates and infected with *L.* *monocytogenes* F2365 InlB corrected pAD-P*plcA*-GFP (right panels) or *L.* *monocytogenes* F2365 InlB corrected pAD-P*hly*-GFP (left panels). Host cells were infected for 6 h and fixed. GFP is shown in green. Actin (red) and nuclei (blue) were labeled with phalloidin conjugated to Alexa 546 and Hoechst, respectively. Bars, 5 µM.

To determine whether the reduced virulence of the *L.* *monocytogenes* F2365 PrfA* Δ*plcA* mutant in chicken embryos was related to a decreased ability to infect cells, we quantified the internalization [2 h post-infection (hpi)] and replication (6 hpi) of the F2365 and F2365 PrfA* strains as well as their isogenic *plcA* deletion mutants in avian HD11 macrophages. Deletion of *plcA* did not affect growth of the F2365 or F2365 PrfA* strains in liquid BHI culture media (data not shown). *plcA* expression was important at 2 and 6 hpi in PrfA^WT^ and PrfA* genetic backgrounds for HD11 infection, since lower numbers of F2365 ∆*plcA* and F2365 PrfA* ∆*plcA* strains were detected when compared to F2365 and F2365 PrfA* bacteria, respectively (Figure 3A). Interestingly, the differences of PlcA in cell infection were more significant when expressed in a PrfA* context (Figure 3A), raising the question about the activity of the *plcA* promoter. To address this issue, a transcriptional reporter with the *plcA* promoter fused to GFP was generated in order to assess *plcA* gene expression in the F2365 strain. The GFP reporter was cloned into the integrative plasmid pAD (generating pAD-P*plcA*-GFP) and introduced in single copy in the genome of a F2365 strain in which we previously corrected its native nonsense InlB mutation [7], allowing to assess the regulation of the *plcA* promoter at the single-bacterium level by microscopy upon cell infection. A similar GFP transcriptional reporter was used for *hly* (pAD-P*hly*-GFP) as a control. We next infected HD11 avian macrophages and Jeg-3 human epithelial cells with the F2365 pAD-P*plcA*-GFP or F2365 pAD-P*hly*-GFP strains for 6 h. Intracellular F2365 pAD-P*hly*-GFP was fluorescent at 6 hpi in the cell lines tested, indicating that the *hly* promoter is highly active in a PrfA^WT^ background (Figure 3B). Interestingly, the activity of the *plcA* promoter was undetectable at 6 hpi in a PrfA^WT^ background in the cell lines tested (Figure 3B), or at other infection times tested (2 h and 24 hpi data not shown). These results demonstrate that the *plcA* promoter is less active than the *hly* promoter in *L.* *monocytogenes* F2365 PrfA^WT^ background, and suggest that the absence of role for PlcA during the chicken embryo infection is due to the low expression of the phospholipase in the F2365 WT strain.

## Discussion

Most of the studies performed to evaluate the role of *L.* *monocytogenes* virulence factors have been carried out using evolutionary lineage II strains, which are rarely associated with human epidemics [9]. In the present study we evaluated the contribution of the main toxins encoded by LIPI-1 and LIPI-3 of a *L.* *monocytogenes* strain responsible for a human listeriosis outbreak [15]. In both PrfA^WT^ and PrfA* genetic backgrounds, LLS and PlcB minimally contribute to virulence in chicken embryos infected in the allantoic cavity. This result is valuable since LLS has been associated with infectious potential in human listeriosis cases (always associated with consumption of contaminated food followed by intestinal barrier cross and deep organ colonization) [9, 26]. The absence of a role for LLS in virulence in chicken embryos is in agreement with our previous observation that demonstrated a major role for LLS as a bacteriocin in the intestine during oral and intravenous infection of mice [7, 8]. These results confirm that LLS contribution to virulence is minimal in animal infection models that bypass the intestinal route (as in this chicken embryo model where *L.* *monocytogenes* is inoculated in the allantoic cavity) [7]. Moreover, we show that LLS is controlled independently of PrfA.

It has been long thought that PlcA contribution to virulence is minimal (no defect in escape from a primary vacuole or cell-to-cell spread and only two- to threefold increase in LD_50_ in mice for Δ*plcA* mutants) and that PlcB and LLO are the main molecular tools of *L.* *monocytogenes* mediating vacuolar escape and cell-to-cell spread [6]. The data presented here show that the importance of PlcA in *L.* *monocytogenes* pathogenesis could be more significant than previously thought, with its effect masked until now by the low activity of the *plcA* promoter in the *L.* *monocytogenes* strains and infection models commonly used. Earlier reports using *L.* *monocytogenes* strains carrying a PrfA^WT^ also showed that the level of *plcA* transcripts is lower than those of *hly* during intracellular growth of *L.* *monocytogenes* in bone marrow-derived murine macrophages or Caco-2 cells [27] or when these genes are expressed in a heterologous host like *Bacillus subtilis* [28]. These results indicate that although *plcA* and *hly* promoters are regulated by PrfA, they are active at different levels in a PrfA^WT^ background.

Previous studies performed to evaluate the contribution of PlcA to *L.* *monocytogenes* pathogenesis were performed with the *L.* *monocytogenes* 10403S strain which does not display a PrfA* phenotype [6], preventing it from expressing high amounts of PlcA. The generation of an artificial PrfA* mutation in the *L.* *monocytogenes* lineage II strain EGD-e rendered it more virulent than the WT strain in HeLa, Jeg-3 and RAW264.7 cells, and in a mouse intravenous infection model [10]. Unfortunately, mutations in the PrfA regulated genes in this EGD-e PrfA* strain were not performed to uncover the origin of the hypervirulent phenotype. The PrfA* mutation performed in the present study in the F2365 strain is artificial, but there are cases (like in the lineage II EGD strain) which naturally display a PrfA* mutation leading to constitutive production of LIPI-1 virulence genes [10]. Recently, it was suggested that PrfA becomes gradually activated during the course of infection, with *hly* expression occurring with PrfA at a less active state and *actA* expression only taking place when PrfA is fully activated by glutathione (resembling a PrfA* phenotype, [29, 30]). This highlights the necessity to also test the role of previously identified virulence factors in a PrfA* background to fully appreciate their function.

The biological relevance of the PlcA results obtained using the PrfA* strain should be interpreted in the context of a constitutive activation of PrfA. However, it can be speculated that in a PrfA^WT^ genetic background, wild type PlcA levels might significantly increase in a tissue-specific manner (e.g. phospholipid-rich tissue like the brain) where its function is critical for successful infection. Moreover, it is remarkable that from all characterized food or human *L.* *monocytogenes* isolates, only one strain displays a truncation on *plcA* in its genome [9], suggesting an important role for this virulence factor during infection or in the overall fitness of *L.* *monocytogenes* in nature.

Altogether, the present results show that LLO and PlcA exotoxins play a major role during *L.* *monocytogenes* pathogenesis in the chicken embryo model when the intra-allantoic route is used whilst PlcB and LLS are dispensable. Further research is necessary to decipher the precise contribution to virulence of historic but yet not completely understood PrfA-regulated virulence factors like PlcA.

## Additional files

**Additional file 1.** **Oligonucelotides primers used in this study for pMAD construction.**

**Additional file 2.** **Oligonucleotide primers used in this study for qPCR.**

## References

[1] Cossart P (2011). Illuminating the landscape of host-pathogen interactions with the bacterium *Listeria monocytogenes*. Proc Natl Acad Sci U S A.

[2] Hoelzer K, Pouillot R, Dennis S (2012). Animal models of listeriosis: a comparative review of the current state of the art and lessons learned. Vet Res.

[3] Yin Y, Tian D, Jiao H, Zhang C, Pan Z, Zhang X, Wang X, Jiao X (2011). Pathogenicity and immunogenicity of a mutant strain of *Listeria monocytogenes* in the chicken infection model. Clin Vaccine Immunol.

[4] Vazquez-Boland JA, Kocks C, Dramsi S, Ohayon H, Geoffroy C, Mengaud J, Cossart P (1992). Nucleotide sequence of the lecithinase operon of *Listeria monocytogenes* and possible role of lecithinase in cell-to-cell spread. Infect Immun.

[5] Kocks C, Gouin E, Tabouret M, Berche P, Ohayon H, Cossart P (1992). *L.* *monocytogenes*-induced actin assembly requires the *actA* gene product, a surface protein. Cell.

[6] Smith GA, Marquis H, Jones S, Johnston NC, Portnoy DA, Goldfine H (1995). The two distinct phospholipases C of *Listeria monocytogenes* have overlapping roles in escape from a vacuole and cell-to-cell spread. Infect Immun.

[7] Quereda JJ, Nahori MA, Meza-Torres J, Sachse M, Titos-Jimenez P, Gomez-Laguna J, Dussurget O, Cossart P, Pizarro-Cerda J (2017). Listeriolysin S is a streptolysin S-like virulence factor that targets exclusively prokaryotic cells in vivo. MBio.

[8] Quereda JJ, Dussurget O, Nahori MA, Ghozlane A, Volant S, Dillies MA, Regnault B, Kennedy S, Mondot S, Villoing B, Cossart P, Pizarro-Cerda J (2016). Bacteriocin from epidemic *Listeria* strains alters the host intestinal microbiota to favor infection. Proc Natl Acad Sci U S A.

[9] Maury MM, Tsai YH, Charlier C, Touchon M, Chenal-Francisque V, Leclercq A, Criscuolo A, Gaultier C, Roussel S, Brisabois A, Disson O, Rocha EP, Brisse S, Lecuit M (2016). Uncovering *Listeria monocytogenes* hypervirulence by harnessing its biodiversity. Nat Genet.

[10] Becavin C, Bouchier C, Lechat P, Archambaud C, Creno S, Gouin E, Wu Z, Kuhbacher A, Brisse S, Pucciarelli MG, Garcia-del Portillo F, Hain T, Portnoy DA, Chakraborty T, Lecuit M, Pizarro-Cerda J, Moszer I, Bierne H, Cossart P (2014). Comparison of widely used *Listeria monocytogenes* strains EGD, 10403S, and EGD-e highlights genomic variations underlying differences in pathogenicity. MBio.

[11] Andersson C, Gripenland J, Johansson J (2015). Using the chicken embryo to assess virulence of *Listeria monocytogenes* and to model other microbial infections. Nat Protoc.

[12] Polakowska K, Lis MW, Helbin WM, Dubin G, Dubin A, Niedziolka JW, Miedzobrodzki J, Wladyka B (2012). The virulence of *Staphylococcus aureus* correlates with strain genotype in a chicken embryo model but not a nematode model. Microbes Infect.

[13] Nix EB, Cheung KK, Wang D, Zhang N, Burke RD, Nano FE (2006). Virulence of *Francisella* spp. in chicken embryos. Infect Immun.

[14] Jacobsen ID, Grosse K, Slesiona S, Hube B, Berndt A, Brock M (2010). Embryonated eggs as an alternative infection model to investigate *Aspergillus fumigatus* virulence. Infect Immun.

[15] Linnan MJ, Mascola L, Lou XD, Goulet V, May S, Salminen C, Hird DW, Yonekura ML, Hayes P, Weaver R, Audurier A, Plikaytis BD, Fannin SL, Kleks A, Broome CV (1988). Epidemic listeriosis associated with Mexican-style cheese. N Engl J Med.

[16] Beug H, von Kirchbach A, Doderlein G, Conscience JF, Graf T (1979). Chicken hematopoietic cells transformed by seven strains of defective avian leukemia viruses display three distinct phenotypes of differentiation. Cell.

[17] Arnaud M, Chastanet A, Debarbouille M (2004). New vector for efficient allelic replacement in naturally nontransformable, low-GC-content, gram-positive bacteria. Appl Environ Microbiol.

[18] Nightingale KK, Milillo SR, Ivy RA, Ho AJ, Oliver HF, Wiedmann M (2007). *Listeria monocytogenes* F2365 carries several authentic mutations potentially leading to truncated gene products, including inlB, and demonstrates atypical phenotypic characteristics. J Food Prot.

[19] Quereda JJ, Pucciarelli MG (2014). Deletion of the membrane protein Lmo0412 increases the virulence of *Listeria monocytogenes*. Microbes Infect.

[20] Samba-Louaka A, Pereira JM, Nahori MA, Villiers V, Deriano L, Hamon MA, Cossart P (2014). *Listeria monocytogenes* dampens the DNA damage response. PLoS Pathog.

[21] Archambaud C, Gouin E, Pizarro-Cerda J, Cossart P, Dussurget O (2005). Translation elongation factor EF-Tu is a target for Stp, a serine-threonine phosphatase involved in virulence of *Listeria monocytogenes*. Mol Microbiol.

[22] Balestrino D, Hamon MA, Dortet L, Nahori MA, Pizarro-Cerda J, Alignani D, Dussurget O, Cossart P, Toledo-Arana A (2010). Single-cell techniques using chromosomally tagged fluorescent bacteria to study *Listeria monocytogenes* infection processes. Appl Environ Microbiol.

[23] Gripenland J, Andersson C, Johansson J (2014). Exploring the chicken embryo as a possible model for studying *Listeria monocytogenes* pathogenicity. Front Cell Infect Microbiol.

[24] Ripio MT, Dominguez-Bernal G, Lara M, Suarez M, Vazquez-Boland JA (1997). A Gly145Ser substitution in the transcriptional activator PrfA causes constitutive overexpression of virulence factors in *Listeria monocytogenes*. J Bacteriol.

[25] Quereda JJ, Ortega AD, Pucciarelli MG, Garcia-Del Portillo F (2014). The *Listeria* small RNA Rli27 regulates a cell wall protein inside eukaryotic cells by targeting a long 5′-UTR variant. PLoS Genet.

[26] Cotter PD, Draper LA, Lawton EM, Daly KM, Groeger DS, Casey PG, Ross RP, Hill C (2008). Listeriolysin S, a novel peptide haemolysin associated with a subset of lineage I *Listeria monocytogenes*. PLoS Pathog.

[27] Bubert A, Sokolovic Z, Chun SK, Papatheodorou L, Simm A, Goebel W (1999). Differential expression of *Listeria monocytogenes* virulence genes in mammalian host cells. Mol Gen Genet.

[28] Sheehan B, Klarsfeld A, Msadek T, Cossart P (1995). Differential activation of virulence gene expression by PrfA, the *Listeria monocytogenes* virulence regulator. J Bacteriol.

[29] Reniere ML, Whiteley AT, Hamilton KL, John SM, Lauer P, Brennan RG, Portnoy DA (2015). Glutathione activates virulence gene expression of an intracellular pathogen. Nature.

[30] Reniere ML, Whiteley AT, Portnoy DA (2016). An in vivo selection identifies *Listeria monocytogenes* genes required to sense the intracellular environment and activate virulence factor expression. PLoS Pathog.

