# Supplementary material for: Role in virulence of phospholipases, listeriolysin O and listeriolysin S from epidemic Listeria monocytogenes using the chicken embryo infection model
Source: Vet Res. 2018 Feb 6;49:13. doi: 10.1186/s13567-017-0496-4 (PMC5801685; doi:10.1186/s13567-017-0496-4)
Supplement: Supplementary file 1 — Additional file 1. Oligonucelotides primers used in this study for pMAD construction. [file 13567_2017_496_MOESM1_ESM.docx]

| Name | Sequence 5’-3’ | Purpose of use |
| --- | --- | --- |
| prfa*-a | ctgatcggatccgctaacaattgttgttactgcc | Amplification of ~500 bp fragment in flanking region of *prfA* |
| prfa*-b | gattaaaagttgagaacaaatagagcc | Amplification of ~500 bp fragment in flanking region of *prfA* |
| prfa*-c | ctatttgttctcaacttttaatcctgac | Amplification of ~500 bp fragment in flanking region of *prfA* |
| prfa*-d | gagtcacccgggcaatcacacttgctgctaaag | Amplification of ~500 bp fragment in flanking region of *prfA* |
| prfa*-x | atatctccgagcaaccatcg | Verification of the *prfA** mutation (mapping in reading frame) |
| prfa*-y | agcgccgattgctattattg | Verification of the *prfA** mutation (mapping in reading frame) |
| plca-a | aatgacccgggaaatagagccgagcttcccg | Amplification of ~500 bp fragment in flanking region of *plcA* |
| plca-b | taatcaaaggagggggccattatattattcctacaaaaaaagggttag | Amplification of ~500 bp fragment in flanking region of *plcA* |
| plca-c | aatggccccctcctttgatta | Amplification of ~500 bp fragment in flanking region of *plcA* |
| plca-d | tcgaggtcgacattgcattcacaacttggatatct | Amplification of ~500 bp fragment in flanking region of *plcA* |
| plca-x | tcttgatgccatcaggagtttc | Verification of the Δ*plcA* mutation (mapping in reading frame) |
| plca-y | tgacaggaagaacatcgggtt | Verification of the Δ*plcA* mutation (mapping in reading frame) |
| hly-a | aatgacccggggttttgctcgtcttttaaacgcat | Amplification of ~500 bp fragment in flanking region of *hly* |
| hly-b | gtgttaagcggttttattcttaatttttgggtttcactctccttctacat | Amplification of ~500 bp fragment in flanking region of *hly* |
| hly-c | aaaaattaagaataaaaccgcttaacac | Amplification of ~500 bp fragment in flanking region of *hly* |
| hly-d | tcgaggtcgactcatcattatcagtcaagtaaccat | Amplification of ~500 bp fragment in flanking region of *hly* |
| hly-x | tttgattagtaatcctaagctgcc | Verification of the Δ*hly* mutation (mapping in reading frame) |
| hly-y | tagggattttattgctcgtgtca | Verification of the Δ*hly* mutation (mapping in reading frame) |
| plcb-a | aatgacccggggctgatttaagagatagaggaac | Amplification of ~500 bp fragment in flanking region of *plcB* |
| plcb-b | ggataagaatatattcctaaatattgacctagaaccacttttttgaactt | Amplification of ~500 bp fragment in flanking region of *plcB* |
| plcb-c | caatatttaggaatatattcttatccac | Amplification of ~500 bp fragment in flanking region of *plcB* |
| plcb-d | tcgaggtcgacgcgactaacataaccgccata | Amplification of ~500 bp fragment in flanking region of *plcB* |
| plcb-x | agtggcgattttacagatgacg | Verification of the Δ*plcB* mutation (mapping in reading frame) |
| plcb-y | ttatatgtgtatgttaatttagaatcaac | Verification of the Δ*plcB* mutation (mapping in reading frame) |
