# Supplementary material for: Role in virulence of phospholipases, listeriolysin O and listeriolysin S from epidemic Listeria monocytogenes using the chicken embryo infection model
Source: Vet Res. 2018 Feb 6;49:13. doi: 10.1186/s13567-017-0496-4 (PMC5801685; doi:10.1186/s13567-017-0496-4)
Supplement: Supplementary file 2 — Additional file 2. Oligonucleotide primers used in this study for qPCR. [file 13567_2017_496_MOESM2_ESM.docx]

| Name | Sequence 5’-3’ |
| --- | --- |
| plcA-RT-PCR-F | tcggatccaaccactaatca |
| plcA-RT-PCR-R | ccgcggacatcttttaatgt |
| hly-RT-PCR-F | cgtccatctatttgccaggt |
| hly-RT-PCR-R | ctccaccattcccaagctaa |
| plcB-RT-PCR-F | ccgagaagggaaatttgaca |
| plcB-RT-PCR-R | tattggcgtgcataggttga |
| mpl-RT-PCR-F | atacgaagggcaatcaggtg |
| mpl-RT-PCR-R | acgcacacagacatcctcac |
| actA-RT-PCR-F | cgaaaacagcgagacaacag |
| actA-RT-PCR-R | aaacccgcatttcttgagtg |
| orfX-RT-PCR-F | aatcgcgttatgttccgtatt |
| orfX-RT-PCR-R | caagacactcaatctcctttgc |
| llsA-RT-PCR-F | tcacaatcatcaaatggctaca |
| llsA-RT-PCR-R | caagaacatgagcaacatcca |
| llsG-RT-PCR-F | gagagagcgcagtttttacaca |
| llsG-RT-PCR-R | tcgttgtttttctccaccag |
| llsH-RT-PCR-F | cccggatattgatgccagta |
| llsH-RT-PCR-R | ggaagttccgaaaaagatgaaa |
| llsX-RT-PCR-F | ttcacatgaatgatggcaca |
| llsX-RT-PCR-R | ttcccaccatctcactacca |
| llsB-RT-PCR-F | ggcaattcaccaatgctagg |
| llsB-RT-PCR-R | tccatttctcttgcctcgtt |
| llsY-RT-PCR-F | acatggagaaactggctgct |
| llsY-RT-PCR-R | caaacatcaattcagctgtgg |
| gyrA-RT-PCR-F | gcgatgagtgtaattgttg |
| gyrA-RT-PCR-R | atcagaagtcatacctaagtc |
